# Epidemiology of Q fever in humans in four selected regions, Spain, 2016 to 2022

**DOI:** 10.2807/1560-7917.ES.2024.29.27.2300688

**Published:** 2024-07-04

**Authors:** Daniel Cifo, Rosa M Estévez-Reboredo, David González-Barrio, Isabel Jado, Diana Gómez-Barroso

**Affiliations:** 1 Carlos III Health Institute – National School of Public Health (ISCIII – ENS), Madrid, Spain; 2 UNED – ENS Mixed Research Institute (IMIENS), Spain; 3 Carlos III Health Institute – National Centre of Epidemiology (ISCIII – CNE), Madrid, Spain; 4 Carlos III Health Institute – National Microbiology Centre (ISCIII – CNM). Department of Bacteriology. Majadahonda, Madrid, Spain; 5 Consortium for Biomedical Research in Epidemiology and Public Health (CIBERESP), Madrid, Spain

**Keywords:** Q fever, Bacterial zoonoses, Spain, Spatial analysis, Infectious disease outbreaks, One Health, Public health surveillance

## Abstract

**Background:**

Q fever is a bacterial zoonosis caused by *Coxiella burnetii.* Spain has the highest number of notified human cases in Europe. Small ruminants are a key reservoir for the pathogen, transmission from animals to humans is usually airborne.

**Aim:**

We aimed at exploring temporal and spatial epidemiological patterns of sporadic and outbreak cases of Q fever in four Spanish regions with the highest number of notified cases.

**Methods:**

We extracted data on Q fever cases in the Canary Islands, Basque Country, La Rioja and Navarre between 2016 and 2022 from the Spanish National Epidemiological Surveillance Network. We calculated standardised incidence ratios (SIR), spatial relative risks (sRR) and posterior probabilities (PP) utilising Besag-York-Mollié models.

**Results:**

There were 1,059 notifications, with a predominance of males aged 30–60 years. In Basque Country, La Rioja and Navarre area, 11 outbreaks were reported, while no in the Canary Islands. A seasonal increase in incidence rates was observed between March and June. In the Canary Islands, elevated sRR was seen in La Palma, Gran Canaria, Lanzarote and Fuerteventura. In Basque Country, La Rioja and Navarre area, the highest sRR was identified in the south of Biscay province.

**Conclusion:**

Goats were the main source for humans in outbreaks reported in the literature. Seasonal increase may be related to the parturition season of small ruminants and specific environmental conditions. Local variations in sRR within these regions likely result from diverse environmental factors. Future One Health-oriented studies are essential to deepen our understanding of Q fever epidemiology.

**Key public health message**

**What did you want to address in this study and why?**

Q fever is a bacterial disease caused by *Coxiella burnetii* affecting humans and animals. Since 2016, Spain has notified the highest number of Q fever cases in Europe. However, our understanding of how the disease occurs and spreads is still limited. To learn more, we conducted a study in the four regions of Spain where Q fever cases are most common: the Canary Islands, Basque Country, La Rioja and Navarre.

**What have we learnt from this study?**

Q fever was more frequent among males aged 30–60 years. Cases tended to increase each year between March and June, which aligns with the time when sheep and goats give birth and environmental conditions may be more favourable. Goats seem to be the main source of the disease in these areas.

**What are the implications of your findings for public health?**

Our study suggests that local climate conditions and livestock activities may affect transmission of the bacterium. Therefore, our findings further support the necessity of adopting a One Health-approach for effective preventive and control measures, including surveillance.

## Introduction

Q fever is a bacterial zoonosis caused by *Coxiella burnetii* affecting humans and many animal species worldwide. The disease is considered underreported [1]. In livestock, the infection is often unnoticed, except during abortion waves [2]. In humans, the acute form of Q fever commonly manifests either as atypical pneumonia or febrile acute hepatitis [1]. Approximately 1-5% of the patients may develop a chronic form of the illness, endocarditis or vascular infection, with a mortality rate of approximately 15% [3]. Diagnosis of Q fever is mainly based on serological testing or PCR [1].

*Coxiella burnetii* is an obligate intracellular pathogen with two antigenic forms, phase I and phase II, relevant for diagnosis of the acute and chronic forms. The bacterium has two morphological forms: the large cell variant (LCV) and the small cell variant (SCV) [1]. The LCV is a metabolically active form replicating within host cells, while the SCV is a dormant form found in the environment, also named spore-like due to its capacity of resisting harsh conditions and persisting in soils or food [1,4].

The bacterium has been isolated from over 100 animal species [5], including at least seven tick species [6]. The main reservoirs are sheep, goats and cattle. Infected females (domestic and wild animals) excrete large amounts of bacteria during parturition or abortion via faeces, vaginal mucus, aborted fetuses, placenta, urine and milk [7]. The main route is through inhalation of contaminated particles. This can occur both close to and far from the source, as the bacterium can move up to 30 km with the wind [8]. Foodborne transmission is considered less common [4]. Human-to-human transmission has been observed in certain instances but is considered rare. Other modes of transmission, such as vertical or tick-borne have not been documented in humans so far [1].

A large Q fever outbreak occurred in the Netherlands between 2007 and 2010, with 4,000 notified cases and more than 40,000 estimated cases, demonstrating that Q fever can pose a substantial public health threat [9]. In Europe, Spain has had the highest number of annually notified human cases since 2016 (0.7 cases per 100,000 population) [10]. Case series have been described in most of the Spanish territory [11]. Since 2015, Q fever has been a notifiable disease in humans in Spain. Notifications are sent to the Spanish National Network of Epidemiological Surveillance (RENAVE). According to the latest reports, the autonomous communities with the highest notification rates between 2016 and 2018 were the Canary Islands, Basque Country, La Rioja and Navarre [12].

Our main objective was to explore the epidemiology of Q fever and to determine possible risk factors in four regions of Spain where the disease is endemic. We also evaluated the spatial distribution in these regions at a municipal level.

## Methods

We extracted data from the RENAVE database on notified Q fever cases in humans in the Canary Islands, Basque Country, Navarre and La Rioja, between 1 January 2016 and 31 December 2022. The data were extracted on 28 April 2023 by the Spanish System for Epidemiological Surveillance (SIVIES), a software that provides access to the nationwide surveillance databases of the RENAVE. A large outbreak with 108 notified cases in Basque Country occurring between 2020 and 2021 [13] was not included in our study due to incomplete data at the time of extraction. Data from the entire country are presented in Supplementary Table S1, S2 and S3.

### Geographical scope

**Figure 1:**
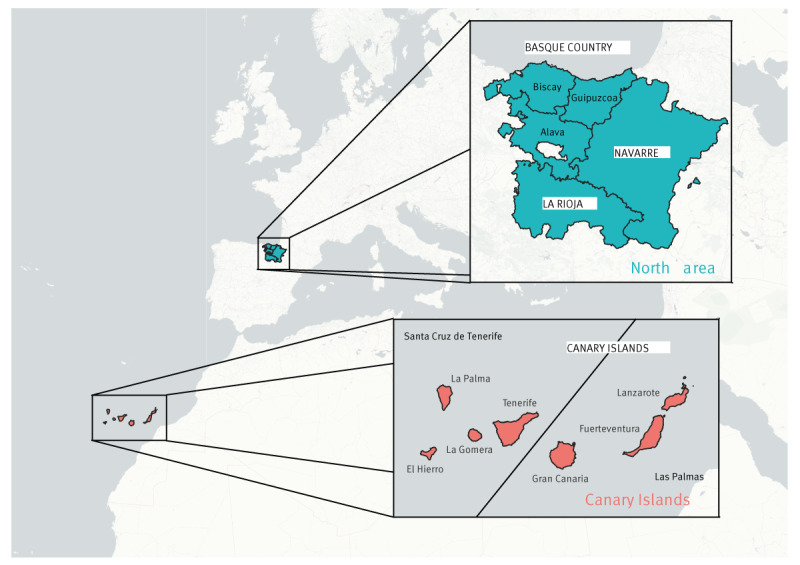
Map presenting regions included in a study of epidemiology of Q fever in humans, Spain, 2016–2022

The Canary Islands, situated in the Atlantic Ocean and part of Macaronesia, are located 100 km west of the coast of Morocco. The regions of Basque Country, La Rioja and Navarre are situated in the north of the Iberian Peninsula, near the Pyrenees Mountains and the Bay of Biscay (Figure 1). These three regions were treated as a single area, called the north area in this paper.

### Case definition and study variables

The case definition was based on the European Union decision [14], covering clinical, epidemiological and laboratory criteria (Box). Probable cases fulfilled the clinical criteria with an epidemiological link. Confirmed cases had both the clinical and the laboratory criteria. Outbreaks were defined as two or more Q fever cases with an epidemiological association.

Box **Criteria for categorisation of Q fever cases**

**Clinical criteria:**

A person who has at least one of the following:

• Fever

• Pneumonia

• Hepatitis

**Laboratory criteria:**

At least one of the following:

• Isolation of *Coxiella burnetii* from a clinical specimen

• Detection of *Coxiella burnetii* nucleic acid in a clinical specimen

• *Coxiella burnetii* specific antibody response (IgG or IgM phase II)

**Epidemiological criteria:**

At least one of the following two epidemiological links:

• Exposure to a common source

• Animal to human transmission

### Epidemiological description

We studied sociodemographic data (age and sex) in each area. We calculated frequencies, percentages and incidence rates (IR) stratified by sex and age groups of 5 years measured as cases per 100,000 person-years. To calculate the IR, we used resident population estimates per region from the Spanish National Institute for Statistics (INE) as of 1 January each year of the study. We included probable and confirmed cases in the analyses.

For outbreaks, we collected data on region, date and exposures. The location of the case was determined by the municipality where the infection was contracted and if unknown, the municipality of the residency.

### Temporal evolution

We studied changes in the monthly IR by sex and plotted the monthly IR to provide an exploratory evaluation of possible temporal trends or seasonality. The date of symptom onset was considered as the case date. If unavailable, the closest registered date (case notification date or hospitalisation date) was used.

### Spatial analysis

We used indirect standardisation methods to calculate the age-standardised incidence ratios (SIR) per municipality. The population of the Canary Islands and the north area was split into age groups of 5 years in the years between 2016 and 2022.

Using a Bayesian Poisson regression model proposed by Besag-York-Mollié [15], we smoothened the SIR values to obtain spatial relative risks (sRR) for each municipality, as a measure of a relative risk adjusted by a spatial component accounting for the cases observed in adjacent municipalities. We calculated posterior probabilities (PP) for each municipality to assess the credibility of the sRR. We considered PP > 0.8 in the municipalities as significant. These three measures were visualised through choropleth maps.

We used the statistical software R version 4.3.0 (https://www.r-project.org/) and Microsoft Excel 16.0 to calculate basic epidemiological measures and R-INLA package for estimations of sRR and PP. To create the maps, we used open-source QGIS in the version 3.24.3 (https://www.qgis.org/en/site/).

## Results

### Epidemiological description and outbreaks

Between 2016 and 2022, 15,017,716 person-years were observed in the Canary Islands and 590 cases were notified. The IR was estimated to 3.93 per 100,000 person-years. One fatality was notified in a female (case-fatality ratio (CFR): 0.17%). Of these 590 cases, 508 (86.1%) were confirmed, 434 (73.6%) in males. The IR was 5.84 per 100,000 person-years for males and 2.07 for females.

In the north area with 22,228,977 person-years observed, 469 cases were notified, resulting in an IR of 3.12 per 100,000 person-years. Two fatalities were notified in males (CFR: 0.42%). Of the 469 cases, 285 (60.8%) were confirmed and 329 (70.1%) were males. The IR was 3.03 per 100,000 person-years for males and 1.30 for females (Table).

**Table:** Annual incidence rate (per 100,000 population) of Q fever, by age group and sex, in selected regions of Spain 2016–2022ª

Age group (years)	Canary Islands		North area^b^	
	Male n = 434	Female n = 156	Male n = 329	Female n = 140
< 15	0.39	0.21	0.25	0.59
15–19	2.77	0.54	2.60	0.79
20–24	4.39	0.74	2.71	0.41
25–29	8.42	1.08	3.94	1.34
30–34	7.32	1.56	5.21	2.46
35–39	8.45	2.20	5.38	2.75
40–44	8.30	3.07	**5.40**	2.24
45–49	8.39	3.30	3.71	1.92
50–54	**9.22**	3.96	3.71	1.16
55–59	8.29	4.56	2.88	1.93
60–64	5.82	3.37	2.85	1.89
65–69	4.27	2.14	1.66	0.46
70–74	2.47	1.27	1.15	0.83
75–79	1.99	1.63	1.31	0.00
≥ 80	2.12	0.26	2.51	0.50
Total	4.98	1.76	2.46	1.07

^a^ Data from an outbreak in Basque Country 2020–2021 are not included.

^b^ North area covers Basque Country, La Rioja and Navarre.

Maximum value is presented in bold.

Data about notified cases, crude IR and age-standardised IR for the entire country by region and study year are presented in Supplementary Table S1, S2 and S3.

In the Canary Islands, no Q fever outbreaks were reported. In the north area, 104 cases (22.2%) were linked to at least 11 outbreaks.

**Figure 2:**
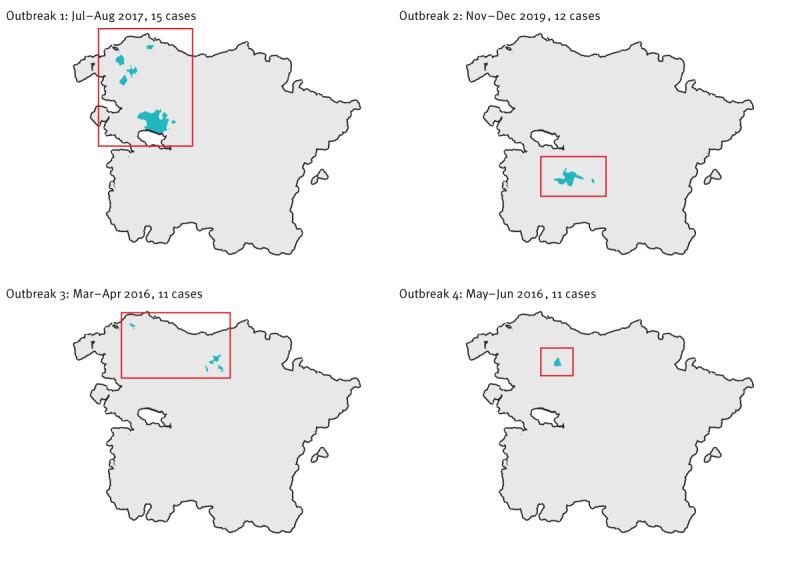
Geographical location, date and number of cases of four largest reported Q fever outbreaks in the north area, Spain, 2016–2017 (n = 49)^a^

^a^ North area covers Basque Country, La Rioja and Navarre.

Of the outbreaks included in the dataset, the largest one with 15 notified cases occurred in Basque Country July–August 2017, with no exposure factors identified. Another outbreak with 12 notified cases occurred in La Rioja November–December 2019 in a tourist apartment near a goat farm in the municipality of Jalón de Cameros. Two additional outbreaks, each with 11 cases, were notified in 2016, both in Basque Country and associated with exposure to ruminant farms. Figure 2 shows the location of the cases in these outbreaks.

Between 2016 and 2019 in the Canary Islands, 94 cases were notified in 2016, 97 in 2017, 92 in 2018 and 111 in 2019. However, during the coronavirus disease 2019 (COVID-19) pandemic years, the number of notified cases declined: 76 cases were notified in 2020, 51 in 2021 and 70 in 2022.

In contrast, more variation was seen in the north area in the pre-pandemic years, with 93 cases in 2016, 116 cases in 2017, 69 cases in 2018 and 92 cases in 2019. During the pandemic, the decline in notified case numbers was more pronounced in the north area than in the Canary Islands, with only 17 and 15 cases notified in 2020 and 2021. In 2022, 76 cases were recorded.

**Figure 3:**
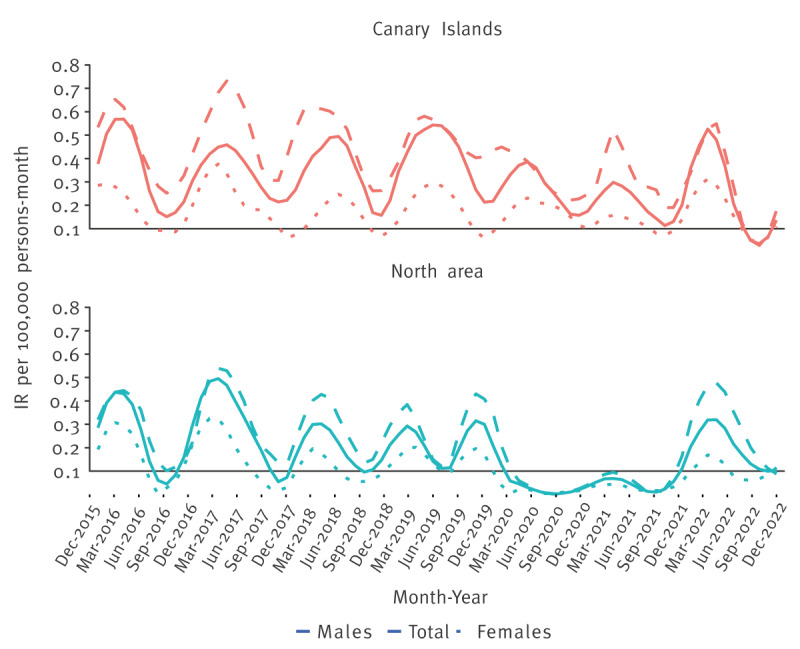
Monthly incidence rate (per 100,000 population) of Q fever, by sex, in selected regions of Spain, 2016–2022ª^,b^

^a^ Data from an outbreak in Basque Country 2020–2021 are not included.

^b^ North area covers Basque Country, La Rioja and Navarre.

The monthly IR analysis (Figure 3) revealed a potential seasonal distribution of Q fever in both areas. Most notifications were between March and June. In the north area, the IR peak appeared earlier than in the Canary Islands, also during the COVID-19 pandemic.

### Spatial analysis

**Figure 4:**
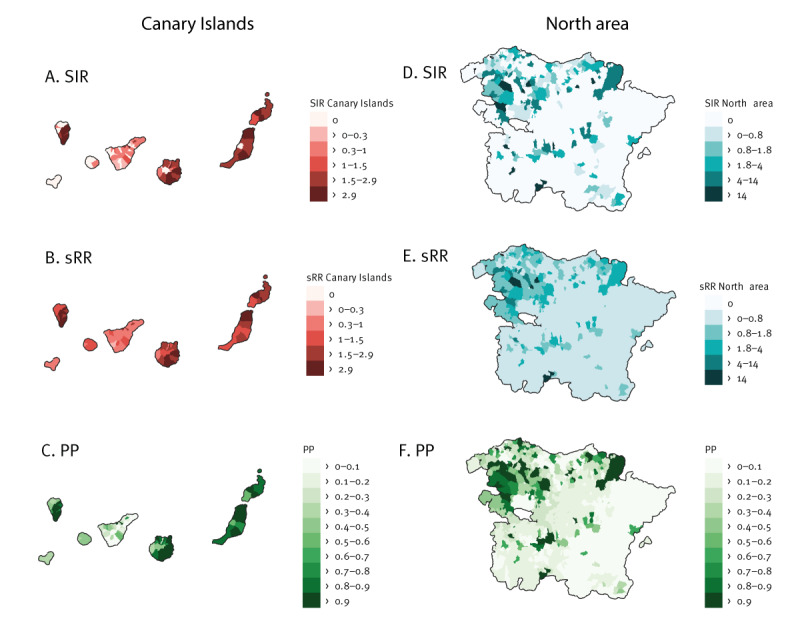
Standardised incidence ratio, spatial relative risk and posterior probability of Q fever, by region, in selected regions of Spain, 2016–2022ª^, b^

PP: posterior probabilities for each municipality to assess the credibility of the sRR; SIR: age-standardised incidence ratio per municipality; sRR: spatial relative risk.

^a^ Data from an outbreak in Basque Country 2020–2021 are not included.

^b^ North area covers Basque Country, La Rioja and Navarre. Spain.

Figure 4A depicts the municipal distribution of SIR in the Canary Islands. Among the seven islands, four were notably affected: the eastern islands of Fuerteventura, Lanzarote and Gran Canaria and the western island of La Palma. The sRR shows a similar distribution pattern highlighting differences in risk between the islands (Figure 4B). In many municipalities of these islands, the PP were > 0.8, indicating a high level of credibility (Figure 4C).

In the north area, the distribution of SIR was heterogeneous, as shown in Figure 4D. Most municipalities with high incidence were concentrated to Basque Country, with a few hotspots in La Rioja and Navarre. We identified a region covering an area of approximately 2,250 km^2^ that extends from the northern part of the Alava province to the southern part of the Biscay province displaying a high risk (Figure 4E). Additionally, we identified some other areas with elevated risk, mainly around Logroño, the capital city of La Rioja and the westernmost part of the Pyrenees Mountains in Navarre. Figure 4F illustrates the PP in these identified high-risk areas, which are primarily > 0.8.

## Discussion

Spain has the highest number of notified Q fever cases in Europe, but the epidemiological knowledge of the disease is still limited [10,12]. The endemicity has been confirmed in seroprevalence studies in the Basque and the Canarian populations [16,17].

The highest Q fever IR was in males aged 30–60 years, as in previously published studies from Spain [11]. This could be related to an occupational exposure, which is well documented in the literature. In a meta-analysis, the seroprevalence of Q fever in slaughterhouse workers was estimated to be higher than 25% [18]. Furthermore, agriculture workers in the United States were more likely to be seropositive than occupants of other domains [19]. Similar associations have been observed in Europe [20]. Nevertheless, it is important to note that further analysis will be necessary to completely assess occupation as a risk factor.

However, particularly due to the capability of *C. burnetii* SCV variant to persist in the environment and be transported over long distances, a proportion of the infected individuals may not have been exposed to known risk factors such as direct contact with livestock. This was seen in the Netherlands in the 2007-2010 outbreak, where some cases lived in the vicinity of goat farms without direct contact with animals [9]. Furthermore, most cases included in a Spanish systematic review were from urban environments [11]. This may represent a limitation in comprehending risk factors for Q fever.

In our study, 11 outbreaks were notified, all in the north area. Although we had limited information on exposures associated with the outbreaks, a prevailing factor was the connection, occupational or not, with ruminant farms. At least three outbreak reports within the same time frame and geographical area have been previously documented.

One of the outbreaks included in our study (July–August 2017) occurred among workers of a courier pet company with 10 confirmed and six probable cases, with an attack rate of 25% [21]. The possible source of the infection was contaminated dust within pet holding facilities, likely after a transport of miniature goats. The probability of the involvement of other animal species was considered low based on the *C. burnetii* genotype determination.

Another outbreak occurring between December 2016 and February 2017 was associated with a dairy goat farm in Biscay following a large abortion wave in does. The first cases were a group of seven farm workers. Weeks later, an additional four cases were notified in a group of visitors to the farm. The attack rate was 78% among workers and 31% among visitors [22].

The most recently notified outbreak spanned between December 2020 and October 2021 involving 108 notified cases among visitors to a natural cave in the southern Biscay province. *Coxiella burnetii* DNA was detected in faecal, dust and aerosol samples inside the cave and in dust samples from 44 farms located within a buffer of 7 km [13]. Unfortunately, data about this outbreak were not available in the database at the time of extraction.

Previously, sheep were considered the main reservoir in the provinces of the north area, based on seroprevalence studies [23]. However, the outbreaks notified in the last 10 years have been linked to goats. This is a relevant observation given that data from the National Livestock Survey indicates that sheep population in these regions is up to seven times greater than the goat population [24]. No outbreaks were reported in the Canary Islands during the study period. The reasons for this, including under reporting and difficulties in detecting a common exposure to extensive farms, need to be elucidated.

The numbers of Q fever notifications showed a consistent trend over the years, except for 2020 and 2021. The decline in notifications during these years could be attributed to the COVID-19 pandemic effects on the surveillance system, which may have also impaired the declaration and registration of outbreaks, regionally and nationally. However, this decline was not uniform as in the Canary Islands the notifications were sustained compared with the north area. In 2022, notifications were at pre-pandemic levels.

An interesting feature is a possible seasonal pattern, which may require further analysis. Except for the pandemic years, monthly IR consistently peaked between March and June. In some Spanish studies from cases [11], including the north area [25] and the Canary Islands [26], most notifications were in spring. The parturition period of small ruminants coincides with these months, which could facilitate the spread of these bacteria [27]. In addition, the increase of airborne particles, microbial and non-microbial, in the air, mainly due to favourable environmental conditions, could further increase the risk.

In the municipal-level spatial model of the Canary Islands, variations in sRR between different islands was apparent. On three of the westernmost islands El Hierro, La Gomera and Tenerife, lower sRR were estimated in contrast to the easternmost islands Fuerteventura, Lanzarote and Gran Canaria. These differences may stem from climatological and weather-related factors. Wind or vegetation may have an impact on bacterial dispersion [8]. The Canary Islands are characterised by a diverse landscape, the western islands are subtropical and humid, and the eastern islands are arid and influenced by dust storms from the Sahara Desert. Although the precise influence of these factors on the epidemiology of Q fever on the islands is unclear, divergent winds of the archipelago have different airborne bacterial composition [28].

Similarly, in the north area, we identified differing sRR values across the territory. The highest sRR and PP values are found between the Basque provinces of Biscay and Alava. In this rural, mountainous area, which includes the Gorbeia Natural Park, several of the aforementioned outbreaks have occurred [13,21]. Livestock farming in Basque Country is prominent with small-scale sheep and goat farms managed in a traditional manner, sometimes lacking resources, which may hinder implementation of preventive measures such as hygiene practices or testing. A significant association was found in the Biscay province between a positive PCR result for *C. burnetii* from farm dust samples and reported outbreaks in humans [25]. These same areas were flagged as high risk in our study. *Coxiella burnetii* DNA has also been detected in deer and wild boar spleens in Biscay [29].

An interesting fact about these two endemic regions is their shared characteristic of high human population density [30], which can lead to a concentration of farms, due to limited available space. The provinces of Basque Country are the smallest in Spain and have abundant valleys and mountains. Likewise, the Canary Islands are a group of relatively small islands with a mountainous terrain. Differences of sRR at the municipal level could also respond to factors related with the livestock industry like the concentration of deliveries in specific months or the urbanisation of grazing areas.

We recognised some limitations. The quality of information provided by regional surveillance systems depends on the level of alertness and the resources and priorities set by each region. The frequent localisation of notification centres in urban areas may lead to an overrepresentation of cases occurring within urban settings. Moreover, inaccuracies in the location of the acquisition of the infection may arise. The impact of the COVID-19 pandemic on surveillance networks may have introduced additional difficulties, including problems in outbreak registration. Focusing on a limited number of regions restricted the geographical scope of the study. Considering plausible factors influencing Q fever transmission, the lack of environmental and climatological data, information on leisure activities, along with related to reservoirs represent additional limitations in risk assessment studies.

## Conclusion

Control measures aimed at reducing Q fever incidence in humans should prioritise a coordinated One Health approach involving surveillance and outbreak management complemented by animal public health measures. In Spain, preventive activities predominantly focus on biosecurity protocols. The Ministry of Agriculture, Fisheries and Food has recently issued a programme that emphasises these measures [31]. Vaccination of livestock is not mandatory but strongly recommended. Additionally, further One Health-orientated epidemiological studies considering the complex interplay between human health, animal health and the environment to develop a comprehensive understanding of Q fever transmission dynamics and risk factors are needed.

## Supplementary Data

253000 Supplementary Material

## References

[1] Eldin C Mélenotte C Mediannikov O Ghigo E Million M Edouard S From Q fever to Coxiella burnetii Infection: a paradigm change. Clin Microbiol Rev. 2017;30(1):115-90. 10.1128/CMR.00045-16 27856520 PMC5217791

[2] Van den Brom R van Engelen E Roest HIJ van der Hoek W Vellema P . Coxiella burnetii infections in sheep or goats: an opinionated review. Vet Microbiol. 2015;181(1-2):119-29. 10.1016/j.vetmic.2015.07.011 26315774

[3] van Roeden SE Wever PC Kampschreur LM Gruteke P van der Hoek W Hoepelman AIM Chronic Q fever-related complications and mortality: data from a nationwide cohort. Clin Microbiol Infect. 2019;25(11):1390-8. 10.1016/j.cmi.2018.11.023 30543852

[4] Barandika JF Alvarez-Alonso R Jado I Hurtado A García-Pérez AL . Viable Coxiella burnetii in hard cheeses made with unpasteurized milk. Int J Food Microbiol. 2019;303:42-5. 10.1016/j.ijfoodmicro.2019.05.010 31132730

[5] González-Barrio D Ruiz-Fons F . Coxiella burnetii in wild mammals: a systematic review. Transbound Emerg Dis. 2019;66(2):662-71. 10.1111/tbed.13085 30506629

[6] Yessinou RE Katja MS Heinrich N Farougou S . Prevalence of Coxiella-infections in ticks - review and meta-analysis. Ticks Tick Borne Dis. 2022;13(3):101926. 10.1016/j.ttbdis.2022.101926 35190334

[7] Roest HJ van Gelderen B Dinkla A Frangoulidis D van Zijderveld F Rebel J Q fever in pregnant goats: pathogenesis and excretion of Coxiella burnetii. PLoS One. 2012;7(11):e48949. 10.1371/journal.pone.0048949 23152826 PMC3494687

[8] Clark NJ Soares Magalhães RJ . Airborne geographical dispersal of Q fever from livestock holdings to human communities: a systematic review and critical appraisal of evidence. BMC Infect Dis. 2018;18(1):218. 10.1186/s12879-018-3135-4 29764368 PMC5952368

[9] Roest HIJ Tilburg JJHC van der Hoek W Vellema P van Zijderveld FG Klaassen CHW The Q fever epidemic in the Netherlands: history, onset, response and reflection. Epidemiol Infect. 2011;139(1):1-12. 10.1017/S0950268810002268 20920383

[10] European Food Safety Authority European Centre for Disease Prevention and Control . The European Union One Health 2021 Zoonoses Report. EFSA J. 2022;20(12):e07666. 36524203 10.2903/j.efsa.2022.7666 PMC9745727

[11] Alende-Castro V Macía-Rodríguez C Novo-Veleiro I García-Fernández X Treviño-Castellano M Rodríguez-Fernández S Q fever in Spain: Description of a new series, and systematic review. PLoS Negl Trop Dis. 2018;12(3):e0006338. 10.1371/journal.pntd.0006338 29543806 PMC5871012

[12] Instituto de Salud Carlos III (ISCII). Informe epidemiológico sobre la situación de la Fiebre Q en España. Años 2019, 2020 y 2021. [Epidemiological report on the situation of Q fever in Spain. Years 2019, 2020 and 2021]. Madrid: ISCII; Oct 2022. Spanish. Available from: https://www.isciii.es/QueHacemos/Servicios/VigilanciaSaludPublicaRENAVE/EnfermedadesTransmisibles/Documents/archivos%20A-Z/Fiebre_Q/Fiebre%20Q%20INFORME%2019-21%20final.pdf

[13] Hurtado A Zendoia II Alonso E Beraza X Bidaurrazaga J Ocabo B A Q fever outbreak among visitors to a natural cave, Bizkaia, Spain, December 2020 to October 2021. Euro Surveill. 2023;28(28):2200824. 10.2807/1560-7917.ES.2023.28.28.2200824 37440349 PMC10347893

[14] European Commission (EC). Commission implementing decision 2018/945 of 22 June 2018 on the communicable diseases and related special health issues to be covered by epidemiological surveillance as well as relevant case definitions. Brussels: EC; 22 Jun 2018. Available from: https://eur-lex.europa.eu/eli/dec_impl/2018/945/oj

[15] Besag J York J Mollié A . Bayesian image restoration, with two applications in spatial statistics. Ann Inst Stat Math. 1991;43(1):1-20. 10.1007/BF00116466

[16] Bolaños M Santana OE Angel-Moreno A Pérez-Arellano JL Limiñana JM Serra-Majem L Seroprevalence of infection by Coxiella burnetii in Canary Islands (Spain). Eur J Epidemiol. 2003;18(3):259-62. 10.1023/A:1023342624475 12800952

[17] Sanzo JM García-Calabuig MA Audicana A Dehesa V . Q fever: prevalence of antibodies to Coxiella burnetii in the Basque country. Int J Epidemiol. 1993;22(6):1183-8. 10.1093/ije/22.6.1183 8144303

[18] Woldeyohannes SM Gilks CF Baker P Perkins NR Reid SA . Seroprevlance of Coxiella burnetii among abattoir and slaughterhouse workers: A meta-analysis. One Health. 2018;6:23-8. 10.1016/j.onehlt.2018.09.002 30302365 PMC6175780

[19] Walsh MG . Assessing Q fever in a representative sample from the United States population: identification of a potential occupational hazard. Epidemiol Infect. 2012;140(1):42-6. 10.1017/S0950268811000227 21371363

[20] Groten T Kuenzer K Moog U Hermann B Maier K Boden K . Who is at risk of occupational Q fever: new insights from a multi-profession cross-sectional study. BMJ Open. 2020;10(2):e030088. 10.1136/bmjopen-2019-030088 32041851 PMC7045227

[21] Alonso E Eizaguirre D Lopez-Etxaniz I Olaizola JI Ocabo B Barandika JF A Q fever outbreak associated to courier transport of pets. PLoS One. 2019;14(11):e0225605. 10.1371/journal.pone.0225605 31765433 PMC6876792

[22] Álvarez-Alonso R Basterretxea M Barandika JF Hurtado A Idiazabal J Jado I A Q fever outbreak with a high rate of abortions at a dairy goat farm: Coxiella burnetii shedding, environmental contamination, and viability. Appl Environ Microbiol. 2018;84(20):e01650-18. 10.1128/AEM.01650-18 30076194 PMC6182892

[23] Ruiz-Fons F Astobiza I Barandika JF Hurtado A Atxaerandio R Juste RA Seroepidemiological study of Q fever in domestic ruminants in semi-extensive grazing systems. BMC Vet Res. 2010;6(1):3. 10.1186/1746-6148-6-3 20089188 PMC2831013

[24] Ministerio de Agricultura, Pesca y Alimentación. Resultados de las Encuestas de Ganado Ovino-Caprino. Noviembre 2022. Results of surveys of sheep and goats. Madrid: Ministerio de Agricultura, Pesca y Alimentación; Nov 2022. Spanish. Available from: https://www.mapa.gob.es/es/estadistica/temas/estadisticas-agrarias/resultados_nov2022_ovino-caprinod_tcm30-644163.pdf

[25] Zendoia II Barandika JF Hurtado A López CM Alonso E Beraza X Analysis of environmental dust in goat and sheep farms to assess Coxiella burnetii infection in a Q fever endemic area: Geographical distribution, relationship with human cases and genotypes. Zoonoses Public Health. 2021;68(6):666-76. 10.1111/zph.12871 34240552

[26] Espejo E Gil-Díaz A Oteo JA Castillo-Rueda R García-Alvarez L Santana-Báez S Clinical presentation of acute Q fever in Spain: seasonal and geographical differences. Int J Infect Dis. 2014;26:162-4. 10.1016/j.ijid.2014.06.016 25080353

[27] Espí A Del Cerro A Oleaga Á Rodríguez-Pérez M López CM Hurtado A One Health approach: an overview of Q fever in livestock, wildlife and humans in Asturias (northwestern Spain). Animals (Basel). 2021;11(5):1395. 10.3390/ani11051395 34068431 PMC8153578

[28] González-Martín C Pérez-González CJ González-Toril E Expósito FJ Aguilera Á Díaz JP . Airborne bacterial community composition according to their origin in Tenerife, Canary Islands. Front Microbiol. 2021;12:732961. 10.3389/fmicb.2021.732961 34737729 PMC8563076

[29] González-Barrio D Carpio AJ Sebastián-Pardo M Peralbo-Moreno A Ruiz-Fons F . The relevance of the wild reservoir in zoonotic multi-host pathogens: The links between Iberian wild mammals and Coxiella burnetii. Transbound Emerg Dis. 2022;69(6):3868-80. 10.1111/tbed.14758 36335588

[30] Eurostat. Population structure indicators by NUTS 2 region. Luxemburg: Eurostat; 2024. Available from: https://ec.europa.eu/eurostat/databrowser/explore/all/general?lang=en&subtheme=reg.reg_dem.reg_dempoar&display=list&sort=category&extractionId=demo_r_pjanind2

[31] Ministerio de Agricultura, Pesca y Alimentación. Procedimiento ante una comunicación de sospecha de fiebre Q en una explotación de rumiantes y/o comunicación de un brote en personas. [Procedure for reporting suspected Q fever in a ruminant farm and/or reporting an outbreak in people]. Madrid: Ministerio de Agricultura, Pesca y Alimentación; Sep 2023. Spanish. Available from: https://www.mapa.gob.es/es/ganaderia/temas/sanidad-animal-higiene-ganadera/procedimientofrenteafiebreqenrumiantessept2023_tcm30-659891.pdf

