# Supplementary material for: Epidemiology of Q fever in humans in four selected regions, Spain, 2016 to 2022
Source: Euro Surveill. 2024 Jul 4;29(27):2300688. doi: 10.2807/1560-7917.ES.2024.29.27.2300688 (PMC11225260; doi:10.2807/1560-7917.ES.2024.29.27.2300688)
Supplement: Supplementary Material [file 23-00688_CIFO_Supplement.pdf]

SUPPLEMENT 1: Q FEVER SURVEILLANCE INFORMATION IN SPAIN

Disclaimer: This supplementary material is hosted by *Eurosurveillance* as supporting information alongside the article *Epidemiology of Q fever in humans in four selected regions, Spain, 2016 to 2022* on behalf of the authors, who remain responsible for the accuracy and appropriateness of the content. The same standards for ethics, copyright, attributions and permissions as for the article apply. Supplements are not edited by *Eurosurveillance* and the journal is not responsible for the maintenance of any links or email addresses provided therein.

Supplementary table S1:
Total notified cases.
Q Fever. By year and region. Spain (2016-2022).
Shading: regions included in the study

Region	2016	2017	2018	2019	2020	2021	2022
Andalucía	76	62	75	71	45	47	93
Aragón	4	13	23	8	12	33	4
Asturias	0	0	0	0	0	0	0
Baleares	0	0	0	0	0	0	0
Canarias	94	97	92	111	76	51	70
Cantabria	1	2	2	0	0	4	2
Castilla y León	4	13	16	17	9	11	14
Castilla-La Mancha	4	8	12	8	3	6	0
Cataluña	0	0	0	0	0	18	38
Ceuta	0	0	0	0	0	0	0
Comunidad Valenciana	31	64	50	71	10	21	27
Extremadura	3	4	4	9	19	8	21
Galicia	6	5	7	11	14	5	8
La Rioja	3	26	15	14	3	1	2
Madrid	15	34	22	23	14	18	35
Melilla	0	1	1	0	0	0	0
Murcia	0	0	0	0	1	3	15
Navarra	8	7	15	16	7	13	13
País Vasco	82	82	39	62	7	1	61

Supplementary table S2:
Crude incidence rate per 100,000 persons-years.
Q Fever. By year and region. Spain (2016-2022).
Shading: regions included in the study.

Region	2016	2017	2018	2019	2020	2021	2022
Andalucía	0.91	0.74	0.89	0.84	0.53	0.55	1.09
Aragón	0.31	0.99	1.76	0.61	0.90	2.49	0.30
Asturias	0.00	0.00	0.00	0.00	0.00	0.00	0.00
Baleares	0.00	0.00	0.00	0.00	0.00	0.00	0.00
Canarias	4.47	4.60	4.32	5.15	3.49	2.35	3.21
Cantabria	0.17	0.34	0.34	0.00	0.00	0.68	0.34
Castilla y León	0.16	0.54	0.66	0.71	0.38	0.46	0.59
Castilla-La Mancha	0.20	0.39	0.59	0.39	0.15	0.29	0.00
Cataluña	0.00	0.00	0.00	0.00	0.00	0.23	0.49
Ceuta	0.00	0.00	0.00	0.00	0.00	0.00	0.00
Comunidad Valenciana	0.63	1.30	1.01	1.42	0.20	0.42	0.53
Extremadura	0.28	0.37	0.37	0.84	1.79	0.76	1.99
Galicia	0.22	0.18	0.26	0.41	0.52	0.19	0.30
La Rioja	0.95	8.24	4.75	4.42	0.94	0.31	0.63
Madrid	0.23	0.52	0.33	0.35	0.21	0.27	0.52
Melilla	0.00	1.16	1.16	0.00	0.00	0.00	0.00
Murcia	0.00	0.00	0.00	0.00	0.07	0.20	0.98
Navarra	1.25	1.09	2.32	2.45	1.06	1.97	1.96
País Vasco	3.75	3.74	1.77	2.81	0.32	0.05	2.76

Supplementary table S3:
Age-standardized incidence rate per 100,000 persons-years.
Q Fever. By year and region. Spain (2016-2022).
Shading: regions included in the study.
Standard population: Total population of Spain.

Region	2016	2017	2018	2019	2020	2021	2022
Andalucía	1.14	1.33	1.30	1.25	1.04	1.01	1.48
Aragón	0.06	3.44	2.89	1.21	1.25	3.43	0.39
Asturias	0.00	0.00	0.00	0.00	0.00	0.00	0.00
Baleares	0.00	0.00	0.00	0.00	0.00	0.00	0.00
Canarias	5.51	5.49	6.83	5.94	3.99	5.52	5.04
Cantabria	4.28	2.07	0.70	0.00	0.00	0.92	0.49
Castilla y León	0.13	0.73	0.97	1.21	0.18	0.35	1.21
Castilla-La Mancha	0.22	0.28	0.59	0.42	1.02	1.96	0.00
Cataluña	0.00	0.00	0.00	0.00	0.00	0.16	0.66
Ceuta	0.00	0.00	0.00	0.00	0.00	0.00	0.00
Comunidad Valenciana	0.97	1.73	1.57	2.17	0.39	0.69	0.66
Extremadura	0.21	0.08	0.41	0.67	3.03	2.34	4.99
Galicia	0.07	0.70	0.91	0.75	0.66	0.21	0.46
La Rioja	0.00	11.05	4.51	5.67	0.51	0.52	0.60
Madrid	0.67	0.87	0.67	0.47	0.36	0.43	0.73
Melilla	0.00	9.36	1.18	0.00	0.00	0.00	0.00
Murcia	0.00	0.00	0.00	0.00	0.07	0.13	1.82
Navarra	0.60	1.39	8.00	4.18	1.17	2.01	1.91
País Vasco	4.40	6.33	1.90	3.07	0.10	0.02	6.16
